# MARVEL, a Tool for Prediction of Bacteriophage Sequences in Metagenomic Bins

**DOI:** 10.3389/fgene.2018.00304

**Published:** 2018-08-07

**Authors:** Deyvid Amgarten, Lucas P. P. Braga, Aline M. da Silva, João C. Setubal

**Affiliations:** ^1^Departamento de Bioquímica, Instituto de Química, Universidade de São Paulo, São Paulo, Brazil; ^2^INRA, UMR 1347, Agroécologie, Dijon, France; ^3^Biocomplexity Institute of Virginia Tech, Blacksburg, VA, United States

**Keywords:** phage, virus, microbiome, machine learning, random forest

## Abstract

Here we present MARVEL, a tool for prediction of double-stranded DNA bacteriophage sequences in metagenomic bins. MARVEL uses a random forest machine learning approach. We trained the program on a dataset with 1,247 phage and 1,029 bacterial genomes, and tested it on a dataset with 335 bacterial and 177 phage genomes. We show that three simple genomic features extracted from contig sequences were sufficient to achieve a good performance in separating bacterial from phage sequences: gene density, strand shifts, and fraction of significant hits to a viral protein database. We compared the performance of MARVEL to that of VirSorter and VirFinder, two popular programs for predicting viral sequences. Our results show that all three programs have comparable specificity, but MARVEL achieves much better performance on the recall (sensitivity) measure. This means that MARVEL should be able to identify many more phage sequences in metagenomic bins than heretofore has been possible. In a simple test with real data, containing mostly bacterial sequences, MARVEL classified 58 out of 209 bins as phage genomes; other evidence suggests that 57 of these 58 bins are novel phage sequences. MARVEL is freely available at https://github.com/LaboratorioBioinformatica/MARVEL.

## Introduction

In the past few decades, our understanding of microbial life has been profoundly changed by techniques of environmental sampling and high-throughput sequencing (Rappé and Giovannoni, 2003; Handelsman, 2004; DeLong, 2009). The uncultured majority of Bacteria and Archaea is slowly being revealed and so is the largely unknown universe of their viruses (Yutin et al., 2018). Viruses are the most abundant biological entities on Earth, outnumbering bacteria and archaea in the oceans by a factor of at least 10, perhaps 100 (Bergh et al., 1989). The majority of environmental viruses infects bacterial hosts and are therefore termed bacteriophages or simply phages. They have been shown to be important drivers of biogeochemical cycles on Earth (Roux et al., 2016), as well as key players in directing and originating bacterial diversity (Falkowski et al., 2008; Koskella and Brockhurst, 2014; Braga et al., 2018).

Isolation is the gold standard for characterizing and assessing phage diversity, and many new phages are isolated every year from diverse environments such as oceans, composting, and human sewage, among many others (Sullivan et al., 2003; Kumari et al., 2009; Amgarten et al., 2017). However, isolation of viruses is constrained by the requirement of cultivable bacterial isolates as hosts. This hinders the prospection for new phages since the vast majority of the microbial species are uncultivable under laboratory conditions (Solden et al., 2016). In this context, tools for mining viral sequences in large datasets of metagenomic reads and contigs are crucial to retrieve information about novel phage genes and genomes (Rosario and Breitbart, 2011).

Machine learning is a general technique that has gained in popularity in the last few years (Hastie et al., 2009; James et al., 2013). A machine learning algorithm can be trained to recognize a specific biological attribute once a list of example bona fide features is provided. Attributes are commonly referred to as *labels* in supervised learning. A machine learning problem generally consists of trying to assign labels to new objects, given a list of features on which the algorithm was trained. In the case of DNA sequences, commonly used features are GC content, oligonucleotide frequency profiles, and codon usage.

Two popular tools have been developed for prediction of viral sequences in a dataset of DNA sequences (Roux et al., 2015; Ren et al., 2017). VirSorter is a tool for prediction of viral contigs in metagenomic datasets, which uses alignments and similarity search in a database of known viruses (Roux et al., 2015). VirFinder uses a machine learning classifier for the same purpose, but in this case, *k*-mer frequency profiles (frequency of nucleotide words of length *k*) are extracted from contigs and given as input to a previously trained model (Ren et al., 2017). Both tools have good performance and are shedding light into the viral dark matter (Nigro et al., 2017; Hurwitz et al., 2018). However, these tools do not perform well in terms of recall (sensitivity), and therefore they might be missing an overly large fraction of true viral sequences (Roux et al., 2015; Ren et al., 2017).

Here we present MARVEL (Metagenomic Analysis and Retrieval of Viral ELements), a tool for prediction of dsDNA phage sequences in metagenomic bins. MARVEL uses a machine learning approach and three simple genomic features extracted from contig sequences. MARVEL considers a contig sequence to be predicted as part of a previously determined bin (as opposed to treating contigs as isolated objects), seeking to leverage the information that all contigs in a bin are, in principle, part of the same organism.

## Materials and Methods

### Training and Testing Datasets

To build and test MARVEL, the RefSeq microbial dataset was downloaded (January 2018) and only genomes belonging to the Bacteria domain (NCBI txid: 2) and to dsDNA viruses from the *Caudovirales* order (NCBI txid:28883) were selected (this is the *baseline dataset*). Tailed phages were selected at this step as a representative group given that they constitute the majority of viruses present in most environmental samples (Ashelford et al., 2003; Filée et al., 2005; Ackermann, 2007). The baseline dataset was split into two subsets according to the GenBank record date: before January 2016; and January 2016 and thereafter. This time-based division is usually applied in classifiers to simulate the use of the tool on newly isolated sequences (Roux et al., 2015; Ren et al., 2017). We refer to the before-2016 subset as the *training dataset*, and to the 2016-and-later subset as the *testing dataset*. The training dataset has 1,247 phage genomes and 1,029 bacterial genomes, and it was used to train and generate a model for prediction of phage bins. The testing dataset has 335 bacterial genomes and 177 phage genomes. Training and testing datasets have no overlap and are available in MARVEL’s repository page^1^.

Training and testing datasets were further processed to generate mock datasets of contigs with specific lengths. For each fragment length analyzed in this study (2, 4, 8, 12, and 16 kbp), complete genomes were randomly fragmented in 10 contigs of the specified length that may or may not have overlap. Next, contigs belonging to the same organism were clustered to form a simulated bin. This process was performed for both training and testing sets, and the resulting bins were used to train the machine learning algorithm, to asses MARVEL’s performance, and to compare MARVEL against VirSorter and VirFinder.

### Feature Extraction and Classifier Development

As previous studies have shown, genomic features such as DNA *k*-mer profiles and GC content can be strong signals in linking or differentiating genome sequences from bacteria and viruses (Edwards et al., 2016; Ren et al., 2017). However, it is known that phages try to mimic host genome sequences in order to overcome their defenses (Carbone, 2008; Bahir et al., 2009). This causes classifiers based on *k*-mer frequencies to have poor performance in terms of overall accuracy and especially recall. In other words, when one of these classifiers identifies a phage genome, it is almost always correct, but it is likely to miss a majority of new phages present in environmental samples.

Seeking more robust features, we focused our efforts on characteristics related to genome structure and protein translational mechanisms of each organism. Such characteristics require a second layer of information, which may be added by utilization of results from gene prediction programs, such as Prodigal (Hyatt et al., 2010) and GeneMark (Besemer et al., 2001). Therefore, we evaluated phage and bacterial genomes according to six of these genomic features extracted from the baseline dataset of RefSeq complete genomes.

These six features are: average gene length, average spacing between genes, density of genes, frequency of strand shifts between neighboring genes, ATG relative frequency, and fraction of genes with significant hits against the pVOGs database (Grazziotin et al., 2017). *Average gene length* was computed by adding up the length of all predicted CDSs in the genome or in the contigs in a bin (in bp) divided by the total number of predicted CDSs. *Average spacing* was calculated as the mean length in bp of regions between two CDSs. *Density of genes* was calculated as the total number of CDSs divided by genome length measured in kbp. *Frequency of strand shifts* was computed by adding up the number of strand shifts between neighboring genes, and dividing by the total number of CDSs in the genome. *ATG relative frequency* was computed by counting the number of ATG triplets in one of the strands, in all contigs in a bin or in the complete genome, divided by the total number of 3-mers in that sequence (one strand). Finally, each CDS in a genome was searched using HMMscan (Eddy, 2011) against the pVOGs database of viral HMM profiles (Grazziotin et al., 2017) (downloaded in January 2018); a significant hit was noted when the *e-*value was less than or equal to 10^-10^. The number of significant hits was divided by the total number of CDSs to generate the *fraction of genes with significant hits against the pVOGs database*. All values based on predicted CDSs were extracted from GenBank files as available for download in January 2018 (exploratory step) or predicted in simulated fragments by Prodigal (Hyatt et al., 2010) as driven by Prokka (Seemann, 2014).

Using Python Scikit Learn libraries (Pedregosa et al., 2011), we tried different machine learning approaches based on the six features listed above. Specifically: support vector machine (SVM), logistic regression, neural networks, and random forest. Classifiers were evaluated using the training set as well as *k*-fold cross-validation (*k* = 20), with the result that random forest was the best approach for our target prediction. Similar findings about suitability of random forest classifiers in bioinformatics have also been reported (Boulesteix et al., 2012; Zhang et al., 2017).

The relative weight of each feature on a given dataset was calculated by the ID3 implementation of random forest (Quinlan, 1986). Features with low gain of information were removed from the final model, in order to simplify feature extraction in the final version of the tool. The following features were selected as more informative: *gene density, strand shifts*, and *fraction of genes with significant hits against pVOGs database* (see section “Results”). We then extracted these three informative features from a complete training set of 8 kbp simulated bins, and a random forest classifier was trained to be MARVEL’s prediction core. The random forest model was trained with 50 initial tree estimators and leaf pruning; other parameters were set to their default values.

### Tests With Simulated Metagenomic Bins

Simulated bins containing different fragment lengths were generated for genomes of the testing set as previously described to asses MARVEL’s performance. Each test corresponding to a specific fragment length was performed in five randomly sampled replicates of 150 bins (75 bacteria and 75 dsDNA phages). Bins were submitted to MARVEL and predictions were evaluated for true positive rates, specificity, accuracy, and F1 score according to the following standard formulae:

$$\begin{array}{c} TPR=\frac{TP}{TP+FN}\\ SPC=\frac{TN}{TN+FP}\\ ACC=\frac{TP+TN}{TP+FP+TN+FN}\\ F_{1}=\frac{2TP}{2TP+FP+FN} \end{array}$$

Where: *TPR = True positive rate, SPC = Specificity,*

ACC = Accuracy,
TP = True positive count, FP = False positive count,
TN = True negative count, FN = False negative count

### Tests With Real Metagenomic Data From Composting Samples

A dataset of Illumina raw reads from composting samples generated by our group (Antunes et al., 2016) was used to test MARVEL’s performance in real metagenomic data. Five samples were extracted from a composting unit, and whole community DNA was extracted to generate shotgun metagenomic reads; this dataset contains mostly bacterial sequences. Raw reads for all five samples were cross-assembled with metaSpades (Nurk et al., 2017) generating a set of contigs. Metabat2 (Kang et al., 2015) was used for binning with parameters: -m 1500 -s 10000. Other parameters remained with their default values. Resulting bins were evaluated regarding quality and the presence of Bacterial and Archaeal marker genes using CheckM (Parks et al., 2015).

### Pipeline Implementation

MARVEL was coded in Python 3 and uses Prokka (Seemann, 2014) and HMMscan (Eddy, 2011) as important dependencies. As input, MARVEL requires a directory with metagenomic bins in FASTA format; it generates a results directory containing bins predicted as phages. An auxiliary script was made available to generate bins from Illumina paired-end reads using standard tools and methods (Breitwieser et al., 2017).

### Performance Comparison of MARVEL, VirSorter, and VirFinder

Each contig of a simulated bin (10 contigs in total) was individually given as input to VirSorter and VirFinder. For a given tool, an entire bin was considered to be a positive prediction in case at least one of its contigs were predicted as viral (note that in our experimental set-up, there are no bins with both bacterial and viral sequences). A contig was considered viral if predicted in categories I and II for VirSorter, and if the *q*-value was less than or equal to 0.01 for VirFinder. Tests were performed for different fragment lengths and in 30 randomly sampled replicates of 100 bins (50 bacteria and 50 dsDNA phages). Average values of true positive rate, specificity, and accuracy were compared using the Wilcoxon signed-rank test and were considered significant if the *p*-value was less than 0.001.

Running time was measured for all tools using two sets of bins (100 bins averaging 40 kbp and 100 bins averaging 160 kbp) in a standard desktop computer with a 64-bit Intel Core i7-4770 3.4 GHz × 6 CPUs and 8 GB RAM DDR3, running Linux distribution Ubuntu 16.

## Results

**FIGURE 1:**
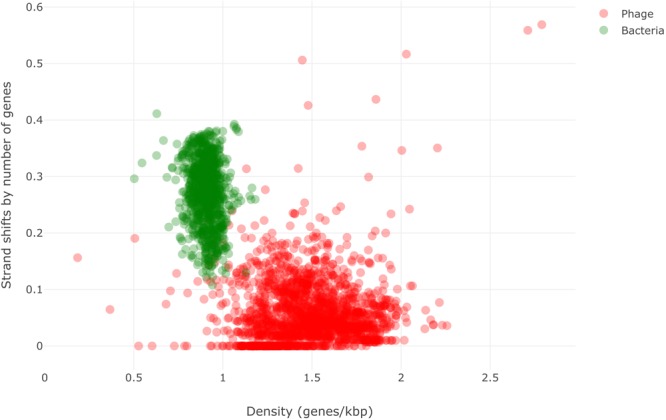
Scatter plot of bacterial and phage genomes using two of the three features as axes: strand shifts by total number of genes and density of genes. Green and red dots represent bacterial and phage genomes, respectively.

As mentioned, we tested six different genomic features; the three best features for our target prediction were *gene density, strand shifts*, and *fraction of significant pVOGs hits*. The relative weights of each feature (based on gain of information) according to the ID3 implementation on both training and testing datasets are: genes density: 0.32, strand shifts: 0.31, pVogs hits: 0.37. **Figure 1** shows results for two of these features on the baseline dataset; numerical results for all three features are shown in **Table 1**. In **Supplementary Figure S1** we also present a PCA analysis of the three selected features.

**Table 1:** Mean values (and respective standard deviations) for three features extracted from the training dataset of dsDNA phage and bacterial genomes.

	Features		
	Gene density (genes by kbp)	Strand shifts by total number of genes	Fraction of pVOGs significant hits
Phage	1.44 (±0.27)	0.07 (±0.05)	0.68 (±0.2)
Bacteria	0.93 (±0.13)	0.24 (±0.08)	0.1 (±0.04)

**FIGURE 2:**
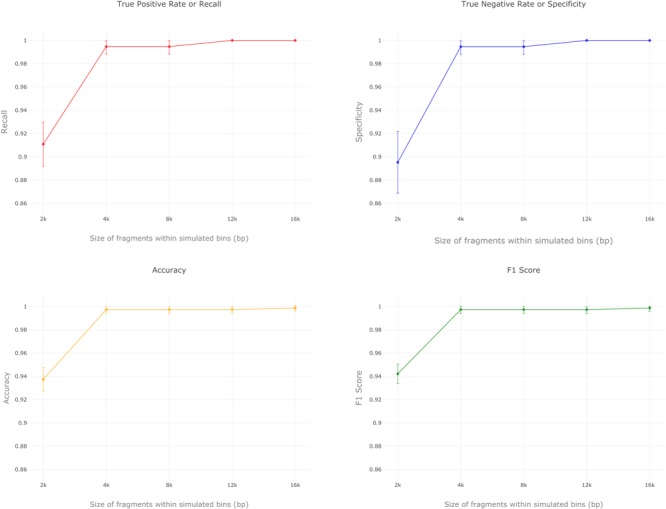
MARVEL’s performance in simulated bins obtained from the testing set of RefSeq genomes. Recall, specificity, accuracy and F1 score are shown for bins composed of different contig lengths.

For a given length, simulated bins were randomly subsampled and given as input to MARVEL in five replicates. Predictions were performed for each simulated bin and results are shown in **Figure 2**. Additional results for k-fold cross validation using both training and testing datasets are presented in **Supplementary Table S1**.

**FIGURE 3:**
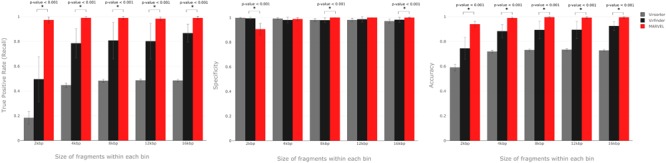
Performance comparison of MARVEL, VirSorter, and VirFinder. Means were compared using Wilcoxon signed-rank test. Standard deviation of 30 replicates are show by error bars. ^∗^ denotes statistically significant difference.

The comparison results between MARVEL, VirSorter, and VirFinder are shown in **Figure 3**. **Table 2** shows running times for each tool with two different sets of bins as input and running on a standard desktop computer.

**Table 2:** Running time for two different set of bins.

	100 bins of ∼40 kbp		100 bins of ∼160 kbp	
	Wall	CPU	Wall	CPU
	time	usage	time	usage
MARVEL	11 m 33 s	17 m 54 s	36 m 33 s	70 m 45 s
VirSorter	10 m 20 s	27 m 21 s	39 m 18 s	140 m 8 s
VirFinder	40 s	40 s	42 s	42 s

*Tests were performed in a desktop computer with a 64-bit Intel Core i7-4770 3.4 GHz × 6 CPUs and 8 GB RAM DDR3, running Linux distribution Ubuntu 16. CPU times are the sum of times spent by each CPU.*

### Identification of Novel Phage Genome Candidates From Composting Samples

Reads from composting samples were assembled and binned, generating 209 bins. These bins were given as input to MARVEL, which classified 58 bins as phage genomes (**Supplementary Table S2**). These 58 bins ranged in length from 10 to 236 kbp (averaging 27 kpb), which are in the expected range of phage genomes (Mahmoudabadi and Phillips, 2018).

We submitted the 209 bins to CheckM. Out of the 58 bins predicted as phages, only one presented bacterial marker genes. This bin contains a CDS predicted to code for a member of the MerR family of transcriptional activators (pfam00376). All other 57 bins were classified by CheckM as “root,” meaning that they had no hits against the set of bacterial marker genes used by CheckM. The potential novelty of the sequences in these 58 bins can be evaluated by observing the number of CDSs in each bin with significant pVOGs hits: the observed range was [14–60]% (**Supplementary Table S2**). The 209 bins used in this test are available at https://github.com/LaboratorioBioinformatica/MARVEL.

## Discussion

**Figure 1** and **Table 1** show that the features chosen can effectively distinguish between bacterial and dsDNA phage sequences. These results suggest that higher gene density and lower rates of strand shift are important phage genomic hallmarks when compared with bacterial genomes. The length of phage genomes is physically constrained by the size of the capsid, which imposes a limited space for genes in the genome (Chirico et al., 2010), favoring increased gene density when compared to bacteria. Evidence supporting very compact phage genomes has also been reported by previous studies (O’Connell, 2005; Roux et al., 2015; Mahmoudabadi and Phillips, 2018). The lower rates of strand shifts can be interpreted as giving phages more efficiency in transcription/translation processes. Such efficiency helps ensure competitive superiority of phage genes over host genes and is essential for phage control of host transcription/translation machinery and cellular resources (Mrázek and Karlin, 1998; Miller et al., 2003). MARVEL’s results have also indicated that the pVOGs database of HMM profiles is comprehensive enough to capture the signal of conserved phage proteins, such as DNA polymerases, helicases, and terminases. These proteins were often identified in the newly discovery genomes, which is in agreement with previous reports from the literature (Rohwer and Edwards, 2002; Comeau et al., 2007). In sum, gene density, strand shifts and pVOGs hits combined as features in a machine learning approach allow more accurate and more sensible prediction of phage genomes compared to other features reported in the literature. Moreover, the relative weights of these three features are approximately the same, suggesting that our model is robust, and should perform well even when a new phage genome has few or no hits to the pVOGs database.

In terms of performance metrics, MARVEL has high F1 scores and accuracy for all bin lengths analyzed, but especially for bins composed of contigs 4 kbp long and longer (**Figure 2**). True positive rates were particularly high for all fragment lengths. As already mentioned, VirFinder and VirSorter do not in general have good recall values, as opposed to specificity, for which their performance is usually very good (Roux et al., 2015; Ren et al., 2017). Altogether, these performance results in simulated data suggest that MARVEL is effectively able to predict dsDNA phage genomes in metagenomic bins.

All three tools have comparable results for specificity in most of the fragment lengths studied, with the exception of 2 kbp-fragments (**Figure 2**). On the other hand, MARVEL’s true positive rates (recall) were significantly higher in all cases (*p*-value < 0.001). MARVEL’s better true positive rates resulted in better overall accuracy compared to the two other tools in all scenarios. Short contigs (2 kbp or less in length) represent a clear limitation, since MARVEL uses CDS predictions as primary information in all three features that we selected. Sequences too short will contain very few or no CDSs, and at least two CDSs are required for calculating the features gene density and frequency of strand shifts. On the other hand, reports in the literature indicate that viral bins are often composed of large contigs, and in some cases contain almost complete viral genomes (Dutilh et al., 2014; Paez-Espino et al., 2017), suggesting that this limitation may not be serious.

The use of MARVEL in one dataset of real data, with contigs having widely varying lengths, yielded promising results, resulting in 57 potentially novel phage sequences.

Upstream processing such as assembly and binning are two major factors that also influence MARVEL’s performance. Chimeric contigs, as well as poorly binned bins may generate noisy data, which will certainly increase the number of erroneous predictions. Therefore, it is important to choose thresholds and parameter values to ensure quality of upstream processing (Mavromatis et al., 2007; Roux et al., 2017). There are several tools available for assembly and binning which generate good quality contigs and bins (Kang et al., 2015; Li et al., 2016; Wu et al., 2016; Nurk et al., 2017). We emphasize, however, that assessing quality of viral bins is not an easy task. CheckM is a tool for assessing marker genes, contamination and completeness of metagenomic bins, but unfortunately only bacterial and archaeal datasets of marker genes are available (Parks et al., 2015).

Our results (**Table 2**) show that MARVEL and VirSorter have comparable running times, while VirFinder is much faster than either. For all programs, wall time was under an hour for what we believe are realistic-sized datasets.

## Conclusion

To our knowledge, MARVEL is the first tool capable to effectively separate metagenomic bins containing dsDNA phage sequences from those containing bacterial sequences. By doing this, it facilitates downstream metagenomic analyses aiming to characterize phage phylogenetic and functional diversity. VirSorter and VirFinder are two excellent tools optimized to analyze single contigs. Although it would be possible to use these tools in a pipeline to generate whole bin predictions, this would certainly require substantial additional work. Furthermore, we present results in simulated data showing significantly better true positive rates and accuracy for MARVEL’s predictions. These improvements were achieved by the implementation and use of three specific genomic features, shown here to be highly suitable for viral sequence prediction.

In its present incarnation, as described here, MARVEL is able to effectively predict tailed phages of the *Caudovirales* order only. Tailed phages constitute the majority of viruses present in most environmental samples, and we believe this fact justifies our choice (Ashelford et al., 2003; Filée et al., 2005; Ackermann, 2007). On the other hand, the features that we used for predictions in this work may not be as effective for viruses in general (Mahmoudabadi and Phillips, 2018). This may be one reason why recall rates in our tests were lower for VirSorter and VirFinder as compared to MARVEL, since those other tools are generic viral sequence finders.

We believe an effective generic viral model would be hard to achieve, given the heterogeneity of viral types and genome structures. Nevertheless, it is our intention to expand MARVEL’s scope to include prediction of other groups of viruses, by obtaining additional models specific to other viral groups. Such models would be available to users as parameter choices in future versions of MARVEL; the program was designed with this objective in mind. We are also working on a module that will seek to provide genome completeness and contamination statistics for each predicted phage genome, similar to what CheckM (Parks et al., 2015) does for bacterial genomes.

## Software Availability

The MARVEL tool, documentation, usage examples, and training and testing datasets are freely available through an online repository^1^.

## Author Contributions

DA conceived, coded, and implemented the tool. JS, AdS, and LB discussed the tool’s design and experimental set-up and results, providing feedback that led to improvements. DA and JS wrote the manuscript. All authors read, revised, and approved the final draft.

## Conflict of Interest Statement

The authors declare that the research was conducted in the absence of any commercial or financial relationships that could be construed as a potential conflict of interest.

## Footnotes

1 https://github.com/laboratoriobioinformatica/MARVEL
