# Supplementary material for: MARVEL, a Tool for Prediction of Bacteriophage Sequences in Metagenomic Bins
Source: Front Genet. 2018 Aug 7;9:304. doi: 10.3389/fgene.2018.00304 (PMC6090037; doi:10.3389/fgene.2018.00304)
Supplement: Supplementary file 3 [file Image_1.pdf]

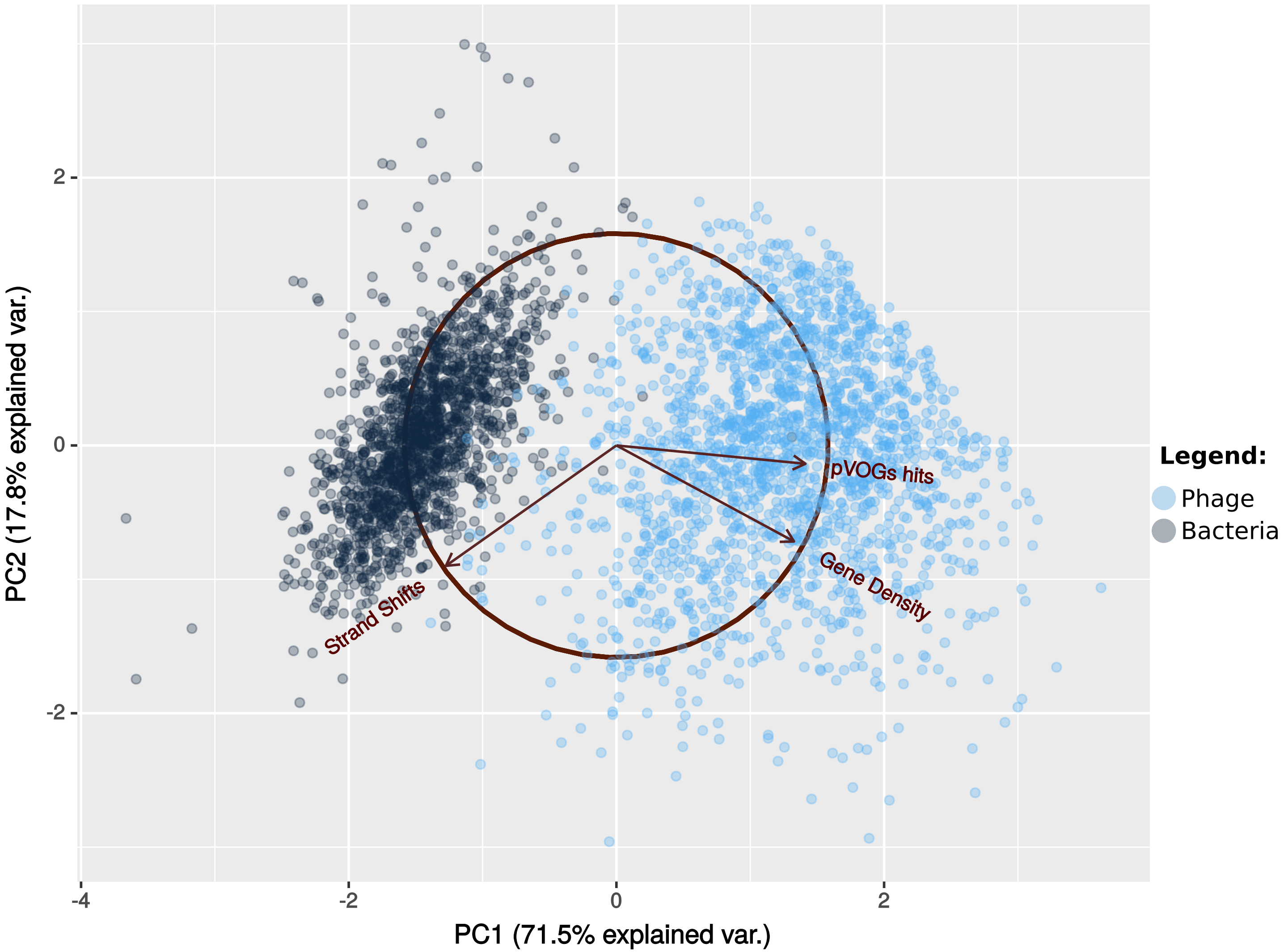

Figure S1: Principal Component Analysis using the three features implemented in MARVEL's Random Forest classifier. The first and second component explain 89% of the variation and each feature contributes proportionaly to the lengh of its vector shown within the circle. Feature values were scaled and centered and PCA analysis was performed with prcomp() function from R.
